# Whole-genome discovery of miRNAs and their targets in wheat (*Triticum aestivum* L.)

**DOI:** 10.1186/1471-2229-14-142

**Published:** 2014-05-22

**Authors:** Fenglong Sun, Guanghui Guo, Jinkun Du, Weiwei Guo, Huiru Peng, Zhongfu Ni, Qixin Sun, Yingyin Yao

**Affiliations:** 1 State Key Laboratory for Agrobiotechnology and Key Laboratory of Crop Heterosis and Utilization (MOE) and Beijing Key Laboratory of Crop Genetic Improvement, China Agricultural University, Beijing 100193, PR China

## Abstract

**Background:**

MicroRNAs (miRNAs) are small, non-coding RNAs playing essential roles in plant growth, development, and stress responses. Sequencing of small RNAs is a starting point for understanding their number, diversity, expression and possible roles in plants.

**Results:**

In this study, we conducted a genome-wide survey of wheat miRNAs from 11 tissues, characterizing a total of 323 novel miRNAs belonging to 276 families in wheat. A miRNA conservation analysis identified 191 wheat-specific miRNAs, 2 monocot-specific miRNAs, and 30 wheat-specific variants from 9 highly conserved miRNA families. To understand possible roles of wheat miRNAs, we determined 524 potential targets for 124 miRNA families through degradome sequencing, and cleavage of a subset of them was validated via 5′ RACE. Based on the genome-wide identification and characterization of miRNAs and their associated target genes, we further identified 64 miRNAs preferentially expressing in developing or germinating grains, which could play important roles in grain development.

**Conclusion:**

We discovered 323 wheat novel miRNAs and 524 target genes for 124 miRNA families in a genome-wide level, and our data will serve as a foundation for future research into the functional roles of miRNAs in wheat.

## Background

Small RNAs, including small interfering RNAs (siRNAs) and microRNAs (miRNAs), are involved in both the transcriptional and posttranscriptional control pathways within nearly every crucial gene cascade in eukaryotic cells [1,2]. MiRNAs are single-stranded non-coding RNAs with sizes most often ranging from 20–22 nucleotides (nt) [3]. MiRNA loci are transcribed by RNA polymerase II into primary miRNA transcripts (pri-miRNAs) that are processed by nuclear RNase III-like enzymes, such as Dicer and Drosha in animals [4] and DICER-LIKE proteins (for example, DCL1) in plants [5]. After being transported to the cytoplasm, miRNAs are incorporated into the RNA-induced silencing complex (RISC) to exert their regulatory functions through cleavage or translation inhibition based on the nearly complementary binding of an mRNA target [6,7]. The examination of miRNAs from various plant species has revealed their possible involvement in organ development, cell differentiation, hormone signaling, biotic and abiotic stress responses, genome maintenance and integrity, and diverse physiological processes [8].

Sequencing of small RNAs is a starting point for understanding their number, diversity, expression and possible roles in plants. Published reports as well as publicly accessible miRNA datasets from different plant species suggest that plant miRNAs are highly complex and abundant. As of June 2013, release 20.0 of the miRBase database contained 7,385 plant miRNA entries, including 337 from Arabidopsis, 713 from rice, 321 from maize, 241 from sorghum, 69 from barley, and 401 from populous (http://www.mirbase.org/) [9]. Sequencing of small RNA populations in plants has established the existence of 16 highly conserved miRNA families with abundant expression, which overwhelmingly regulate the expression of transcription factors that are critical for development or stress responses. Recently developed deep sequencing technologies are uncovering an increasing number of lineage-specific or species-specific miRNAs exhibiting low or tissue-specific expression, which target diverse genes with specialized functions. For example, the *Brassicaceae* family-specific miR824 regulates the expression of *AGAMOUS-LIKE 16*, which plays a role in controlling stomatal density and development in leaves [10]. Therefore, the identification of miRNAs in diverse species has been a major focus in recent years.

Based on miRNA discovery, the miRNAs putatively related to certain tissues development have been identified by deep sequencing technologies. For example, cotton miRNAs show a trend of repression during ovule and fiber development, and this rapid and dynamic change may contribute to ovule and fiber development in allotetraploid cotton [11]. A diverse set of miRNAs and miRNA-like small RNAs have been identified from developing rice grains, some of which are differentially expressed during seed development [12]. Sequencing of sRNA populations from soybean seeds and vegetative tissues has also revealed tissue-preferential expression for certain miRNAs [13]. Interestingly, the recently evolved miR163 displays differences in spatial expression between *Arabidopsis thaliana* and *Arabidopsis arenosa* and in their allotetraploids [14]. These data suggest that species-specific miRNAs and the spatio-temporal regulation of conserved miRNAs play important roles in shaping morphological and developmental variation among related species during evolution [14-16].

MiRNA binding to complementary sequences in target mRNAs regulates eukaryotic gene expression at the post-transcriptional level through mRNA degradation or translational repression [17,18]. Most plant miRNAs induce the degradation of their mRNA targets through precisely cleaving the target sequence between the tenth and eleventh nt from the 5′ end of the miRNA binding site [19]. With the emergence of high-throughput sequencing technologies, degradome analysis or PARE (parallel analysis of RNA ends), which can globally collect 3′ fragments of mRNA targets, is the current choice for validating miRNA targets that are cleaved [20]. Using this method, a large number of target genes have been successfully identified in Arabidopsis [20,21], rice [22], soybean [23] and wheat [24]. These validated targets include transcription factors that play key roles in development and genes involved in a variety of other physiological processes. In addition, miRNA-guided cleavage initiates the entry of primary transcripts into the phase-siRNA biogenesis pathway. For example, *Arabidopsis thaliana* ta-siRNAs form from primary transcripts that are initially targeted and cleaved by the *AGO1–miR173* (*TAS1* and *TAS2*), *AGO1–miR828* (*TAS4*) or *AGO7–miR390* (*TAS3*) complex [25-28]. The tomato 22 nt miR4376 triggers the formation of phase-siRNA from its target *ACA10* gene and may function as a novel layer of a molecular mechanism underlying tomato reproductive growth [29].

Hexaploid wheat, *Triticum aestivum* L. (2n = 6× = 42; genomes AABBDD) is one of the most widely cultivated crops globally due to its high yield and nutritional and processing qualities, providing 20% of the calories consumed by humans (FAO 2011). Previous studies attempted to identify miRNAs associated with development and stress response in wheat by sequencing small RNA population [24,30-36] or by computational strategies [37-39]. For example, our group and Wei et al. identified 43 and 48 wheat miRNA families by sequencing pooled RNAs from leaves, stems, roots and spikes [32,35]. Li et al. also constructed small RNA and degradome libraries leading to identification of 32 miRNAs and their targets from wheat seedlings [33]. From developing grains, around 540 miRNAs putatively associated with grain development were identified [31]. Only a small scale of miRNAs was determined spactial-temporal expression pattern along wheat development, and majority of detected miRNAs were preferentially expressed in certain tissues. However, no whole genome scale miRNA identification and expression comparison among multiple tissues types or developmental stages has been done. In this study, we selected 11 tissues throughout the wheat growth to discover wheat miRNAs in whole genome scale. Moreover, previous study on wheat miRNAs prediction relied on extremely limited wheat genome sequences, and given the larger genome size of wheat, there may be additional miRNAs that have not been identified. Recently, based on the whole-genome shotgun strategy, draft genomes for bread wheat [40], its A-genome progenitor *Triticum urartu* (2n = 14; AA) [41] and its D-genome progenitor *Aegilops tauschii* (2n = 14; DD) [42] have been reported. Furthermore, next-generation sequencing data of flow-sorted individual chromosome arms of wheat were also partly available, provided by International Wheat Genome Sequencing Consortium (IWGSC) (http://www.wheatgenome.org/). Indeed, a recent study predicted miRNAs on wheat chromosome 1AL, 6B and 5D [39,43,44]. In this study, in order to discover wheat miRNAs in whole genome scale by experimental approach, we identified 689 miRNAs from multiple wheat tissues of different developmental stages based on all of the genome sequences available.

Hexaploid wheat is one of the most widely cultivated crops globally due to its high yield and nutritional and processing qualities, providing 20% of the calories consumed by humans (FAO 2011). Despite its agricultural importance of wheat grains, research on the molecular basis of development of wheat grains is limited. Some topics that have been studied include expression profiles of metabolic proteins in endosperm [45] and of mRNA in whole grain [46]. The role of miRNAs during grain development is still unknown, and identification of grain development associated miRNAs could accelerate the progress of wheat improvement and potentially increase its production. In this study, we further screened the miRNAs that were preferentially expressed in wheat grains, which might play important roles in grain development.

## Results

### Distribution of small RNA populations in multiple wheat tissues of different developmental stages

Given the increasingly diverse and significant roles being uncovered for endogenous small RNAs in plant development and stress responses, we first determined the size and composition of the small RNA populations in various wheat tissues of different developmental stages. A total of 11 sequencing libraries were constructed using small RNAs extracted from dry grains (DG), germinating seed embryos (GSE), seedling shoots (SH), seedling leaves (SL), seedling roots (SR), stems in the jointing stage (SJ), 0–5 mm young spikes (YS5), 10–15 mm young spikes (YS15), flag leaves (FL), developing grain of 8 days after pollination (GRA8) and 15 days after pollination (GRA15) in the Chinese Spring wheat cultivar. Solexa high-throughput sequencing generated a total of 118,301,178 sequence reads of 18 to 30 nucleotides in length from these libraries (Table 1). This analysis identified 36,235,609 unique sequence tags, of which 91.5% were singletons, indicating that wheat genome expressed a highly diverse and complex small RNA population.

**Table 1:** Summary of 11 wheat small RNA libraries

Library*	**Distinct reads**	**Total reads**	**Singleton/Distinct (%)**	**20-24 nt/Total (%)**
DG	2,962,991	10,368,525	80.76%	80.80%
GSE	3,368,098	10,831,734	82.58%	79.99%
SH	3,802,991	6,295,504	72.28%	92.29%
SL	1,555,132	4,154,639	77.07%	82.17%
SR	3,060,835	11,368,631	82.97%	72.11%
SJ	7,754,037	13,025,784	84.63%	96.44%
YS5	6,428,180	14,036,832	83.12%	84.49%
YS15	4,841,857	14,355,546	82.13%	88.65%
FL	2,370,690	7,580,952	87.22%	89.60%
GRA8	2,796,755	11,843,843	84.40%	74.79%
GRA15	7,502,165	14,439,188	83.16%	89.23%
Total	36,235,609	118,301,178	91.51%	84.65%

*11 Tissues used to construct libraries in this study. *DG*: dry grain, *GSE*: embryo of germinating seed, *SH*: shoot, *SL*: seedling leaf, *SR*: seedling root, *SJ*: stem in jointing stage, *YS5*: 0-5 mm young spike, *YS15*: 10-15 mm young spike, *FL*: flag leaf, *GRA8*: grains of 8 days after pollination, *GRA15*: grains of 15 days after pollination.

**Figure 1:**
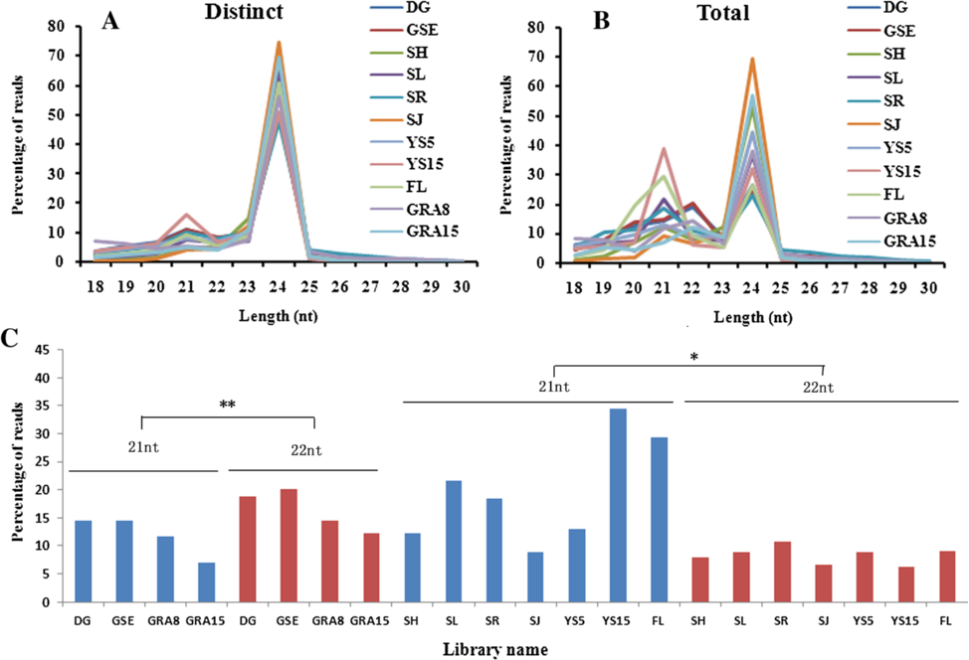
**Length distribution of small RNAs from various tissues of wheat plants. (A)** Distinct reads (only one occurrence per library). **(B)** Total reads. **(C)** Abundance of 21 and 22 nt sRNAs from various tissues of wheat plants. *means abundance of 21 nt miRNA is significantly higher than that of 22 nt in non-seed tissues (P < 0.05). **means abundance of 22 nt miRNA is significantly higher than that of 21 nt in seed related tissues (P < 0.01). DG: dry grain; GSE: germinating seed embryos; SH: shoots; SL: seedling leaves; SR: seedling roots; SJ: stems in the jointing stage; YS5: 0–5 mm young spikes; YS15: 10–15 mm young spikes; FL: flag leaves; GRA8: grains 8 days after pollination; GRA15: grains 15 days after pollination.

We assessed the size distribution of the sequences based on both their total abundances and distinct signatures (Figure 1). Regarding the proportion of distinct sequences of each size, which might represent the extent of the complexity of the wheat small RNA population, we found that 24 nt signatures were prevalent in all of the libraries, whereas 21 nt signatures were less abundant (Figure 1A). These results indicated that 24 nt sRNAs, the majority of which were associated with repeats and transposons, exhibited the highest sequence diversity, consistent with widespread origins of sRNAs within wheat genomes. The overall size distribution patterns of the unique 24 nt sRNAs from the 11 libraries showed striking differences, in that the proportion of distinct 24 nt signatures ranged from 48.0% in seedling roots to 74.5% in stems in the jointing stage. Interestingly, the 10 ~ 15 mm young spikes displayed a large set of distinct 21 nt sRNAs (16.3%) as compared to the other tissues. In terms of total abundance, approximately 85% of the reads were 20-24 nt in length, with 21 and 24 nt representing the major size classes, consistent with being products of cleavage by DCL enzymes (Figure 1B). The most abundant size of the wheat small RNAs was 24 nt, followed by 21 and 22 nt. GRA8 showed significantly higher 24 nt small RNA abundance (57.07%) than average (39.16% ± 14.54%) of all tissues. It was worth noting that dry grain, germinating seed embryos, and 8 and 15 DAP grain significantly produced more 22 nt than 21 nt sRNAs (P < 0.01), whereas in other tissues 21 nt sRNA abundance was significantly higher than 22 nt (P < 0.05) (Figure 1C). Such enrichment of 22 nt sRNAs in grains has been previously observed in maize [47-49] and soybean [13] but not in rice [50,51].

### Genome-wide discovery of wheat miRNAs

Around 539 wheat miRNAs putatively associated with development and stress response have been identified by sequencing small RNA population [24,30-36] (Additional file 1: Table S1). Firstly, we confirmed the presence of a total of 366 known miRNAs from 260 families sharing the exactly same sequences with reported wheat miRNAs in our small RNA sequencing dataset (Additional file 1: Table S1). In order to find novel variants of known miRNAs, we searched small RNAs with 1 or 2 mismatches to known miRNAs in our small RNA sequencing data libraries by use of homolog analysis, which leading to 119 novel variants belonging to known miRNA families (Additional file 2: Table S2).

**Figure 2:**
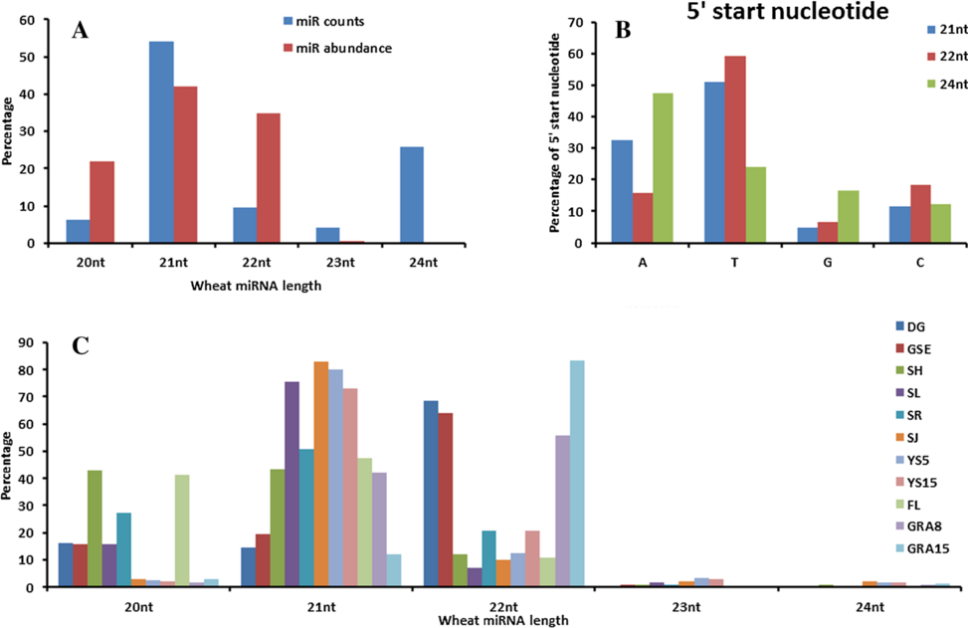
**Length distributions of newly identified miRNAs from various tissues of wheat plants and the preferred 5′ start nucleotides of 21 and 24 nt miRNAs. (A)** Unique counts and abundances of wheat miRNAs of different sizes. **(B)** Abundance of miRNAs of different sizes in various tissues. **(C)** Preferred 5′ start nucleotide for 21 and 24 nt miRNAs. DG: dry grain; GSE: germinating seed embryos; SH: shoots; SL: seedling leaves; SR: seedling roots; SJ: stems in the jointing stage; YS5: 0–5 mm young spikes; YS15: 10–15 mm young spikes; FL: flag leaves; GRA8: grains 8 days after pollination; GRA15: grains 15 days after pollination.

Next, we mapped the rest of small RNAs to wheat genome for the identification of novel wheat miRNAs which showed low homology to known miRNAs, and we found that a total of 28,820,486 small RNA sequences can be perfectly matched to wheat genome. After removing the small RNAs matching to repeat, rRNA and tRNA, the remaining 25,802,718 small RNAs were subjected to miRNA identification. Then, we relied on wheat EST sequences and genome sequences available (See method) as miRNA surrounding sequences in hairpin structure prediction. Finally, sequencing data from 11 libraries supported the identification of 323 wheat novel miRNAs from 276 families (Additional file 2: Table S2). Regarding the unique counts of miRNAs, we found the predominance of 21 nt (54.04%) and 24 nt (25.88%) (Figure 2A), which indicated their origins from wheat genome. When we calculated the abundance of miRNAs, we found that 24 nt miRNAs only accounted for 0.45% of the total abundance, while approximately 98.83% of the miRNAs were 20, 21 or 22 nt in length (Figure 2A), which indicates that a wide range of 24 nt miRNAs originating from diverse loci are expressed at low abundance. The 24 nt long miRNAs has been demonstrated to direct DNA methylation at loci from which they are produced as well as in trans at their target genes and play roles in gene regulation in rice [52]. We further compared the expression patterns of the 21 and 22 nt miRNAs across various tissues, and interestingly, the 22 nt miRNAs showed markedly higher expression levels in seed tissues, including dry grains, germinating seed embryos, and grains 8 and 15 days after pollination, which was quite contrary to the expression pattern of the 21 nt miRNAs (Figure 2C). Next, we analyzed the distribution of the 5′ end nucleotides of the miRNAs. The results revealed that the 24 nt miRNAs showed a strong bias for A as the 5′ terminal nucleotide (Figure 2B), consistent with what is observed in long miRNAs in rice [52], whereas the 21 nt miRNAs exhibited a higher proportion of 5′ ends beginning with U and then A (Figure 2B). These findings provided evidence that the 21 nt and 24 nt miRNAs were generated by different DCLs and that they were specifically sorted into different AGO clade proteins based on hierarchy rules [52].

### Identification of wheat-specific miRNAs and wheat-specific variants for conserved miRNAs

To date, highly and moderately conserved miRNAs have been identified from eudicots to basal plants [53,54]. In this study, we found that all 16 known highly conserved miRNA families were detected in our dataset. Moreover, we also identified 30 wheat-specific variants from 9 highly conserved miRNA families, including miR159, miR160, miR167, miR169, miR171, miR172, miR393, miR396 and miR398 families (Additional file 3: Table S3). These findings indicated that different members of the same miRNA family might evolve at different rates within the same plant species, or most likely associated with the polyploid nature of wheat. To screen the wheat specific miRNAs, we explored the presence of 323 novel wheat miRNAs across Arabidopsis, soybean, rice, maize, sorghum, barley and *Brachypodium*. Among them, 191 were wheat-specific, while orthologs were found for the remainder in other species (Additional file 3: Table S3). We also found 2 monocot specific miRNAs including tae-miR3014b and tae-miR3075 which were conserved among all of the monocots we examined. We analyzed the origin of wheat miRNAs including known and novel miRNAs along the grass evolution, and the results indicated that 55 miRNAs were shared by all monocots indicating their origin from ancient ancestors and 87 miRNAs diverged and retained in wheat, barley, rice and *Brachypodium* after divergence of maize and sorghum from rice. A total of 106 wheat miRNAs were shared with barley but loss in *Brachypodium* and rice (Additional file 3: Table S3). These results indicated that a large number of wheat miRNAs were born at divergence of barley and wheat from rice.

### Transcriptome-wide identification of miRNA targets in wheat through degradome sequencing

To gain insight into the functions of known and novel miRNAs in wheat, miRNA target genes were identified through a degradome sequencing approach. Four libraries, prepared from germinating seeds, seedling leaves, seedling roots and grains collected 8 DAP were constructed for degradome sequencing, and more than 10 M high quality reads were obtained from each library. Because 24 nt miRNAs mainly mediate DNA methylation, only the identified miRNAs with sizes of 20–23 nt were subjected to further target gene analysis in this study.

Based on degradome sequencing, a total of 524 potential targets were identified for 124 wheat miRNA families (Additional file 4: Table S4). The number of predicted targets per miRNA (4.2) was higher in wheat as compared to Arabidopsis (2.9) [20] and rice (2.8) [22], suggesting the existence of additional paralogous and homoeologous genes in this hexaploid species. Among these target genes, 44.7% and 45.8% were regulated by miRNAs at the ORF and 3′ UTR, respectively, and only 9.5% of the genes were targeted in the 5′ UTR. Notably, the cleavage analyses revealed a total of 20 target transcripts that were targeted by more than two distinct miRNAs. For instance, the unigene encoding ATP-sulfurylase 3 was targeted by miR395 within the coding region and by Ta-miR2041, Ta-miR2047 and tae-miR3020 in the 3′ UTR. Although ATP-sulfurylase is similarly targeted by miR395 within its coding region in rice, we did not find similar miRNAs targeting its 3′ UTR, indicating miR395 combined with other miRNAs can target ATP-sulfurylase in a potential wheat-specific pathway.

**Figure 3:**
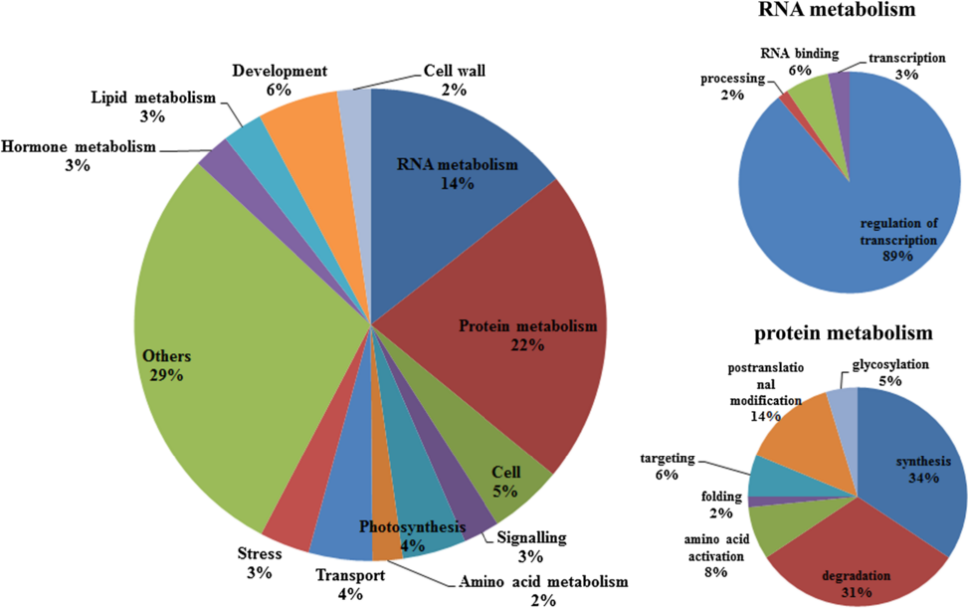
**Functional enrichment of wheat miRNA target genes based on modified MapMan BINs.** The target genes of wheat miRNAs were aligned with known functional categories in Arabidopsis (TAIR9) using blastx, and the best hit was designated as the functional category for the target gene.

To gain a better understanding of the functional roles of the predicted miRNA target genes in wheat, we searched for enrichment in the Mapman molecular function and biological process categories [55]. We found that the identified target genes involved in a wide spectrum of regulatory functions and selected biological processes including RNA metabolism, protein metabolism, hormone metabolism, signaling, development, and transport (Figure 3). Under the category of protein metabolism, a total of 31% and 34% of the miRNA target genes were involved in protein degradation and synthesis, respectively. For RNA metabolism, the genes targeted by miRNAs showed a strong enrichment for transcription factor or transcription regulator activity (89%).

**Figure 4:**
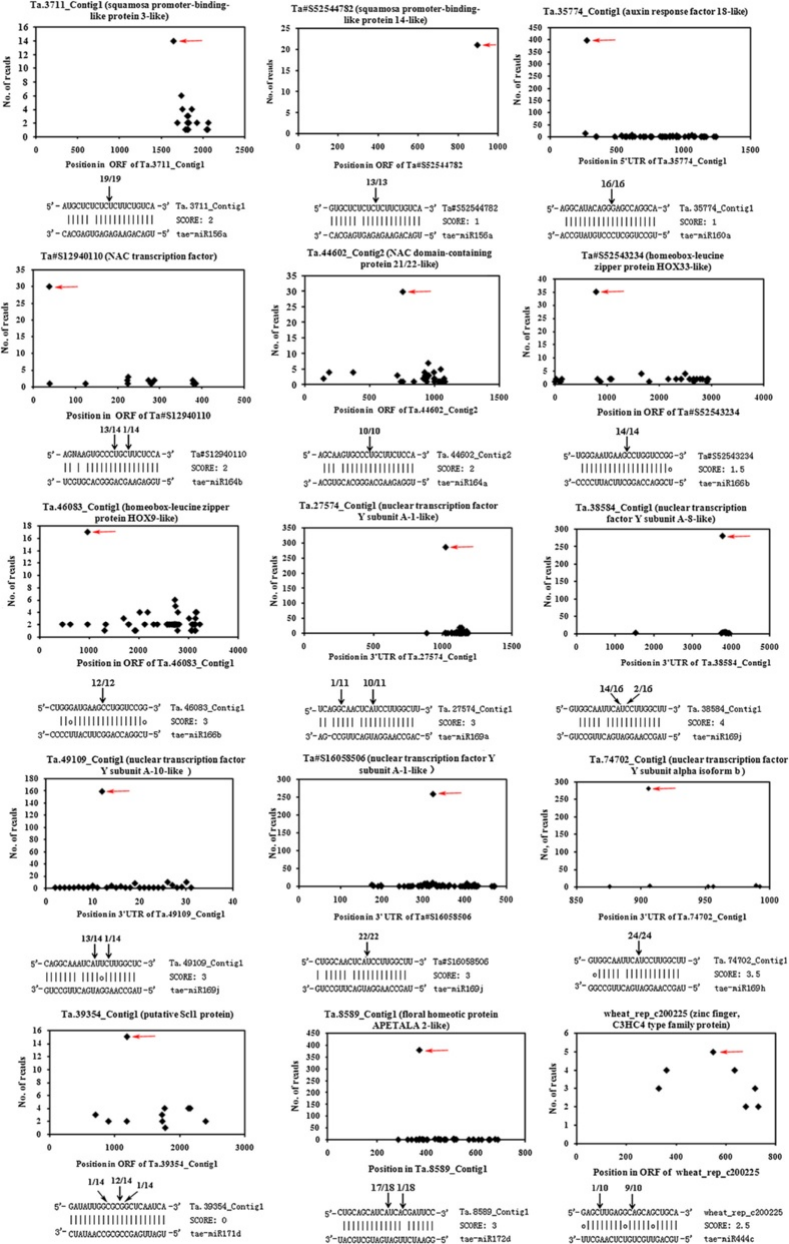
**Confirmed conserved and non-conserved targets of conserved miRNAs determined through degradome sequencing and RNA ligase-mediated rapid amplification of 5′ complementary DNA ends (5′ RACE).** The degradome sequencing of miRNA targets is presented in the form of target plots (t-plots). The signature abundance throughout the length of the indicated transcripts is shown. The red arrows in the t-plots indicate cleavage sites (x-axis) and the detected frequencies (y-axis). The target gene (contig) names are given above the t-plots, with annotations shown in parentheses. miRNA:mRNA alignment along with the cleavage frequencies detected using 5′ RACE. The arrows indicate the 5′ ends of the miRNA-guided cleavage products, and the numbers indicate the ratios of the cleaved products of the total fragments that were sequenced.

It was well established that conserved miRNAs target conserved homologous genes in diverse plant species. In this study, 122 potential target genes for 17 highly or moderately conserved miRNAs were identified. A total of 92 unigenes encoding proteins such as squamosa promoter-binding-like protein, auxin response factors, NAC transcription factors, and MADS-box transcription factors were found to be conserved targets regulated by highly conserved miRNAs in wheat. We also identified 30 non-conserved target unigenes for 11 highly or moderately conserved miRNAs (Additional file 4: Table S4). For instance, in addition to auxin response factors, miR160 was found to target two unigenes encoding FIZZY-RELATED 2-like proteins. Similarly, wheat miR159 was observed to target three unigenes encoding proline-, glutamic acid- and leucine-rich proteins, in addition to the well-conserved MYB family. Based on the degradome sequencing, Proline-, glutamic acid- and leucine-rich proteins gene (Ta#S15902591) was found to be cleaved mainly at the position of 10th and 11th nucleotide of miR159 binding site in two different degradome libraries including GSE and SR (Additional file 5: Figure S1). These findings suggest that at least some conserved miRNAs are regulating nonconserved targets in addition to the well-documented conserved targets. We employed a gene-specific 5′-rapid amplification of cDNA ends (RACE) assay to isolate cleavage remnants for 15 target genes, including 2 *SPL* genes for miR156, 1 *ARF* gene for miR160, 2 *NAC* genes for miR164, 2 HOMEOBOX-LEUCINE ZIPPER genes for miR166, 5 genes encoding nuclear transcription factor Y subunit A proteins for miR169, 1 scarecrow-like protein gene for miR171 and 1 *AP2* gene for miR172, and 1 gene encoding a C3HC4 type zinc finger protein that was regulated by miR444 in wheat. The results of this analysis indicated that the cleavage sites for all 15 of these genes were consistent with the degradome sequencing results (Figure 4). Thus, we provide degradome sequencing and 5′-RACE evidence demonstrating that deeply conserved miRNAs can regulate both conserved and non-conserved targets.

**Figure 5:**
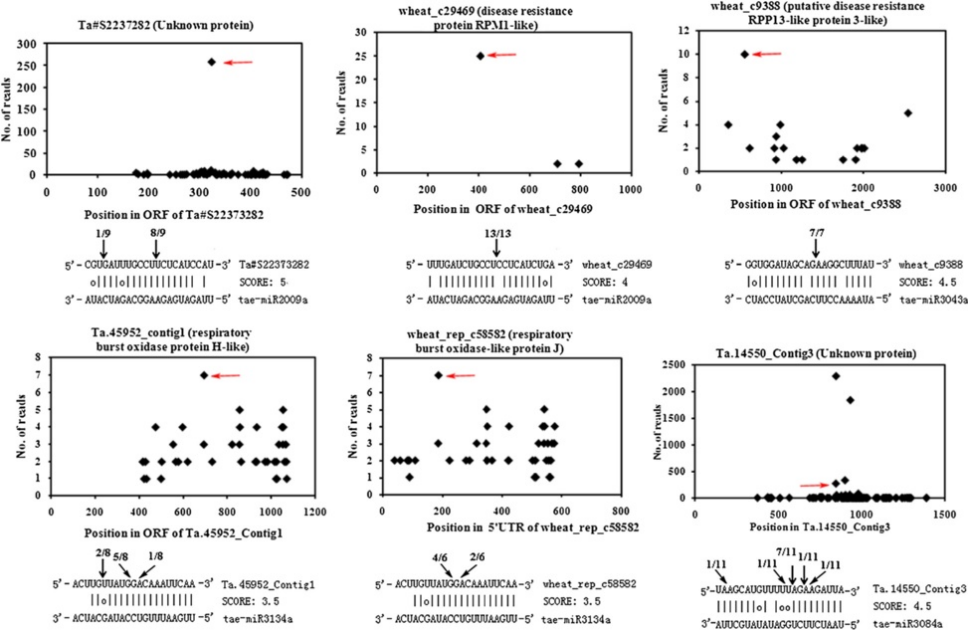
**Confirmed targets of wheat- and barley-specific miRNAs determined through degradome sequencing and RNA ligase-mediated rapid amplification of 5′ complementary DNA ends (5′ RACE).** The degradome sequencing of the miRNA targets is presented in the form of target plots (t-plots). The signature abundance throughout the length of the indicated transcripts is shown. The red arrows in the t-plots indicate cleavage sites (x-axis) and the detected frequencies (y-axis). The target gene (contig) names are given above the t-plots, with annotations shown in parentheses. miRNA:mRNA alignments along with the cleavage frequencies detected using 5′ RACE. The arrows indicate the 5′ ends of miRNA-guided cleavage products, and the numbers indicate the ratios of the cleaved products of the total fragments that were sequenced.

In addition, we focused on the target of wheat-specific miRNAs, and identified 71 targets for 17 wheat-specific miRNAs, which encoded proteins involved a diverse range of processes, including metabolism, development and other physiological processes (Additional file 4: Table S4). To validate the cleavage of these targets, 4 target genes were examined via 5′-RACE. However, none of them were validated as being cleaved at the miRNA binding site. The explanation might be that 1) some species-specific miRNAs lack target genes; 2) the targets of some species-specific miRNAs are expressed at a very low level or in a tissue-specific manner, and they cannot be identified by degradome sequencing; 3) many species-specific miRNAs repress the translation of their target genes, which therefore cannot be determined through degradome sequencing or 5′ RACE methods, which fail to clone the target in the absence of detectable slicing; 4) some miRNAs identified based on small RNA sequencing and prediction of precursor hairpin structure might not be real ones. More evidences are needed to confirm. For example, are they DCL1 dependent? Finally, we identified 150 targets for 61 wheat-barley linage specific miRNAs through degradome sequencing (Additional file 4: Table S4). We validated the cleavage of 6 target genes for 4 wheat-barley-specific miRNAs through 5′-RACE. Among these genes, two unigenes encoding an unknown protein and a disease resistance RPM1-like protein were identified as targets of miR2009a. MiR3134a cleaved two genes, encoding respiratory burst oxidase homolog proteins H and J. MiR3043a targeted unigene encoding disease resistance RPP13-like protein. One unigene encoding an unknown protein was confirmed to be targeted by miR3084a (Figure 5).

### Identification of miRNAs that are preferentially expressed in wheat grains

**Figure 6:**
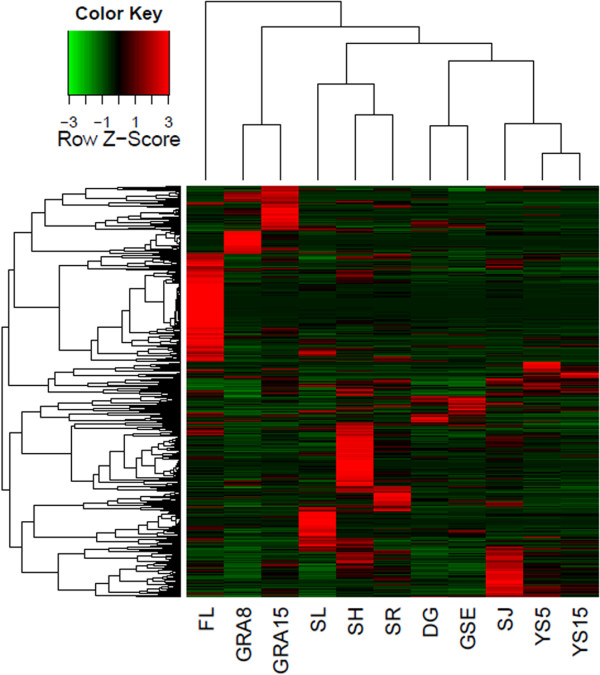
**The complicated expression pattern of wheat miRNAs.** Illumina-sequencing profiles of wheat miRNAs in 11 different tissues. Changes in expression are indicated as upregulation (red) or downregulation (blue). The expression level for each miRNA was normalized to the total mapped reads in each library (RP10M).

In order to screen the miRNAs that are preferentially expressed in grains, we examined the genome-wide transcription profiles of miRNA abundance across 11 different wheat tissues by high throughput sequencing (Additional file 6: Table S5). To visualize broad trends in the temporal expression of the miRNAs, we performed simple hierarchical clustering to generate heatmaps (Figure 6). First, based on the outcome of the tissue classification in heatmaps, tissues that are similar in structure were grouped together, indicating that similar groups of miRNAs were up- or down-regulated in the each tissue type. For example, 8 DAP and 15 DAP grains, 0–5 mm young spikes and 10–15 mm young spikes, dry grains and germinating seed embryos were grouped together. Hierarchical clustering of these tissues by their miRNA expression profiles suggested that the functions of miRNAs can be appreciated based on the biology of the tissues in which it is uniquely expressed.

**Figure 7:**
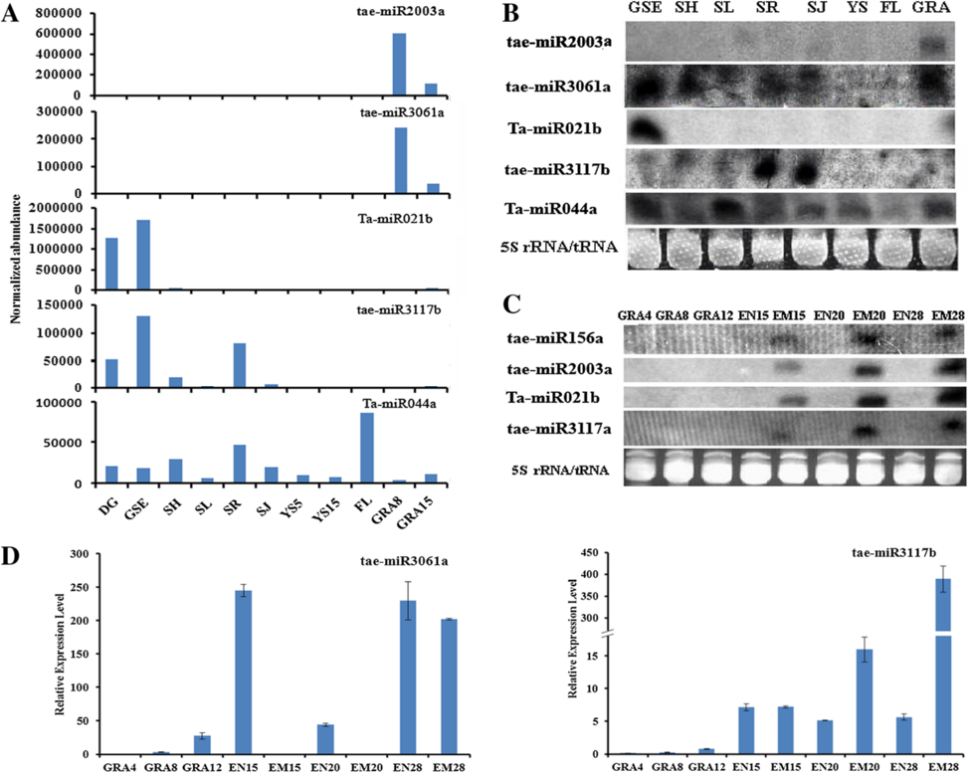
**Northern blotting analysis of intriguing wheat miRNAs across various tissues and the expression of miRNAs abundant in grain at different time points in developing grains. (A)** Normalized expression levels of miRNAs, as revealed by Illumina sequencing reads. **(B)** Validation of the expression miRNA patterns displayed in **(A)** through northern blotting assays. **(C)** Northern blotting analysis of miRNAs in the embryo and endosperm during the development of grains. The 5S rRNA/tRNA bands were visualized via ethidium bromide staining of polyacrylamide gels, which served as a loading control. **(D)** The expression of tae-miR3117b and tae-miR3061a in the embryo and endosperm during the development of grains were determined by RT-PCR. DG: dry grain; GSE: germinating seed embryos; SH: shoots; SL: seedling leaves; SR: seedling roots; SJ: stems in the jointing stage; YS5: 0–5 mm young spikes; YS15: 10–15 mm young spikes; FL: flag leaves; GRA4: grains 4 days after pollination; GRA8: grains 8 days after pollination; GRA12: grains 12 days after pollination; GRA15: grains 15 days after pollination; EN15: endosperm 15 days after pollination; EM15: embryos 15 days after pollination; EN20: endosperm 20 days after pollination; EM20: embryos 15 days after pollination; EN28: endosperm 28 days after pollination; EM28: embryos 28 days after pollination.

The heatmaps helped us to identify 51 miRNAs from 36 families, including miR2003 and miR3061 etc., were specifically expressed in developing grains and the majority of these miRNAs showed similar expression in 8 and 15 DAP grains. A total of 13 miRNAs including miR3002a and miR021b etc. from 12 families accumulated at higher levels in the dry seeds and germinating embryos (Additional file 6: Table S5). MiR3118 showed significantly higher expression level in 15 DAP grains and dry grains compared to other tissues, and one gene encoding glyoxysomal malate dehydrogenase was putatively regulated by miR3118 based on the degradome sequencing. It was reported that during wheat germination the number of glyoxysomes increased, and their associated enzyme including glyoxysomal malate dehydrogenase activities appeared, increasing up to the fifth or sixth day [56]. We speculated that miR3118 negatively regulate glyoxysomal malate dehydrogenase gene during wheat germination. In addition, we also found 4 miRNAs including miR3064, miR3084, miR159b and miR171e were silenced in developing grains. MiR159 negatively regulates the expression of *GAMYB* genes at the posttranscriptional level, which was first identified as a downstream GA signaling target in aleurone cells of barley (*Hordeum vulgare* L.) [57], and *TaGAMYB* also has a pattern of higher expression in wheat developing grains [46]. This result indicated that absence of miR159 in grains leading to higher expression of *TaGAMYB,* which would be consistent with its roles in endosperm grain filling and in the embryo.We further performed Northern blot assays using RNA samples from leaves, roots, stems, shoots, spikes, germinating seed embryos, flag leaves and developing grains to validate the preferential expression the miRNAs in wheat grains (Figure 7). The Northern blot results revealed that tae-miR2003a was expressed specifically in developing grains, and tae-miR3061a showed higher expression in germinating seed embryos, shoots, leaves, roots and stems. Tae-miR021b was expressed specifically in germinating seed embryos, showing strong concordance with the expression profile obtained through Illumina sequencing. Tae-miR3117b exhibited higher expression in stems and roots and relatively lower expression in germinating seeds, shoots, and leaves, different from what was observed in the high-throughput sequencing results (Figure 7A, B). Tae-miR044a showed variable expression pattern across different tissues.The embryos and endosperm of developing grains were not separated during the small RNA library construction and northern blot analysis; therefore, we further examined the expression profiles of grain-abundant miRNAs in embryos and endosperm during grain mature at 4, 8, 12, 15, 20 and 28 days after pollination (DAP) (Figure 7C, D). By Northern blot, we found that tae-miR156a, tae-miR2003a, tae-miR021b, tae-miR3117a specifically accumulated in embryos and all gradually increased from 15 to 28 DAP, with the exception of miR3117a, which showed lower expression in 20 DAP embryo than 15 DAP embryo. The expression of tae-miR3117b and tae-miR3061a were determined by RT-PCR, and the results indicated that tae-miR3117b exhibited the highest expression level in 28 DAP embryo, which was different from tae-miR3117a, even they possessed similar sequences with the exception of two SNPs. In addition, tae-miR3061a mainly appeared in the endosperm and 28 DAP embryo (Figure 7D). These miRNAs were mainly expressed in the late stage of seed development, when starch and storage proteins accumulate and the seeds mature, indicating that these miRNAs might be involved in seed maturation.

## Discussion

### Wheat genome contains a huge set of conserved and wheat-specific miRNAs

Previous studies have reported identification of 510 miRNAs associated with development and stress response in wheat by sequencing small RNA population [24,30-36]. These known miRNAs were identified from certain tissues such as seedlings or developing grains, or from mixed tissues including stems, leaves, roots and spikes. In this study, we extended the identification of wheat miRNAs to 689 and broaden the knowledge of tissues that some miRNAs preferentially expressed. The present study represents the first whole genome scale identification of wheat miRNAs from diverse tissues and the first large scale expression comparison among various tissues. Without a sequenced genome for wheat, it is difficult to map miRNAs to wheat genome sequences and predict potential foldback structures; therefore, these studies have provided only a partial understanding of wheat miRNA population. Recently, based on the whole-genome shotgun strategy, draft genomes for bread wheat [40], its A-genome progenitor *Triticum urartu* (2n = 14; AA) [41] and its D-genome progenitor *Aegilops tauschii* (2n = 14; DD) [42] have been reported, which will facilitate genome-scale miRNA analyses in wheat. In the present study, we systematically annotated a total of 689 miRNAs belonging to 536 families in 11 different tissues based on the draft wheat genome sequences, identifying 69 highly conserved miRNAs and 191 wheat-specific miRNAs at a genome-wide scale, thus significantly increasing the number of known miRNA genes in wheat. This extremely large set of miRNAs is likely associated with the polyploid nature of wheat, which is reasonably consistent with the higher gene numbers, ranging from 94,000 to 96,000, reported in bread wheat than its diploid progenitor and other species such as rice, maize and Arabidopsis. For a given miRNA locus in a diploid, there are three loci in a hexaploid, or more if the locus was duplicated in the diploid or tetraploid progenitor species or duplicated after the allopolyploid event, which might lead to a particularly large set of miRNAs at the genome scale.

### Origins and evolution of wheat miRNAs

Many canonical miRNAs are conserved among moss, eudicots, and monocots, and some regulate conserved targets and display conserved functions among land plants. Our observations regarding 69 highly conserved miRNAs from 16 families provided evidence that these miRNAs are evolutionarily conserved in the plant kingdom. However, we also found multiple wheat-specific variants of conserved miRNAs exhibiting nucleotide substitutions as compared to other species. The divergence of these variants within highly conserved miRNA families might suggest that they have evolved at different rates. Furthermore, through degradome sequencing, we identified and validated a large set of non-conserved targets for the conserved miRNAs, in addition to the conserved target genes. However, the fact that these variations may or may not affect target specificity in wheat although they are wheat-specific, raising the question whether these conserved miRNA variants evolved independently to acquire wheat-specific functions.

The wheat-specific miRNAs identified in this study are particularly interesting because they may function in a species-specific manner in wheat growth and development. In plants, a minority of the annotated miRNA gene families are conserved between plant families, while the majority are family or species specific, suggesting that most known miRNA genes arose relatively recently in evolutionary time [58]. In the present study, several observations indicated that unlike highly conserved miRNAs, species-specific miRNAs are often weakly expressed, processed imprecisely and lack targets. First, we compared the average abundances of highly conserved and wheat-specific miRNAs, which indicated that the average number of reads for highly conserved miRNAs reached 71188.7 RP10M, while that for wheat-specific miRNAs was 3583.6. Box plots show that the normalized expression of highly conserved miRNAs was significantly higher than that of wheat-specific miRNAs (*T* test, P < 0.01) (Additional file 7: Figure S2). Second, we identified 122 target genes for 17 conserved miRNAs and 71 target genes for 17 wheat-specific miRNAs, but we were unable to obtain putative targets for the remainder of the 174 wheat-specific miRNAs, suggesting a lack of or low expression of these target genes. Third, wheat-specific miRNAs regulated fewer targets (4 target genes per miRNA) on average compared to the highly conserved miRNAs (7 target genes per miRNA), further suggesting a lack of targets for the wheat-specific miRNAs.

### Target genes of conserved and wheat-specific miRNAs

The integration of miRNAs in diverse biological networks relies on the conformation of their target genes. Therefore, degradome sequencing has been broadly applied to understand the roles of target gene degradation in transcriptional regulation. In this study, a total of 524 potential targets were identified for 124 putative miRNA families through degradome sequencing, and the cleavage of 19 genes was validated through 5′ RACE. For highly conserved miRNAs, 122 target genes were identified, including 92 targets that are conserved among other species and 30 non-homologous target genes. These non-conserved targets of miRNAs might evolve specific properties and display unique functions in wheat growth and development. In addition, it remains unclear whether the non-conserved targets and conserved targets shared by conserved miRNAs are related to a common biochemical pathway, although they are not homologous genes. It will be worthy to determine whether the non-conserved targets of conserved miRNAs are biologically relevant or merely represent a neutral, accidental event.

There are two outstanding characteristics related to the potential biological functions of the target genes of wheat miRNAs. First, the genes targeted by these miRNAs showed a strong tendency toward displaying transcription factors or transcription regulatory activity. The majority of these targets belongs to conserved ones regulated by highly conserved miRNAs and is involved in diverse aspects of plant growth and development. A large number of target genes were also found to be involved in protein metabolism, among which 38% and 34% were involved in protein degradation and synthesis, respectively. Enrichment of protein synthesis and degradation functions was observed in transition-stage SAMs, and protein synthesis, turnover and balance are required to establish a shoot meristem [59-61]. Therefore, our results might indicate that meristem development is also subject to miRNA regulation through the regulation of target genes responding to protein synthesis and degradation.

### Highly or specifically expressed miRNAs during wheat grain development

The miRNAs found to be highly or specifically expressed during wheat grain development in this study are particularly interesting because wheat grains provide approximately 55% of the carbohydrates consumed by humans [62]. To investigate the roles of small RNAs in grain development and to identify potentially seed-specific small RNAs, several groups have employed high-throughput sequencing technology to sequence small RNA populations from developing seeds in rice [12,50,63], barley [64], maize [49] and soybean [13,23,65]. The obtained sequencing data suggest that rice miR1428e_3p is highly expressed in grains and cleaves two SbRK1b kinases, which play a role in regulating starch accumulation based on their expression in the endosperm and aleurone [12]. In addition, Arabidopsis miRNAs including miR160, miR166, and miR319 inhibit the expression of differentiation-promoting transcription factors such as *ARF17, CNA, PHB, PHV, and TCP4* to enable proper embryonic patterning [66]. The presence of a large set of miRNA molecules in the developing seeds from various species provides some indication that many processes that occur during seed development are under the control of miRNA regulation.

In this study, a total of 51 miRNAs from 36 families were found to be specifically expressed in developing grains, among which 28 miRNAs were wheat specific, indicating these miRNAs might be involved in wheat grain development in a wheat-specific manner. We also found a number of genes associated with grain development that serve as miRNA targets, such as gamma-gliadin and late embryogenesis abundant protein. We found a number of grain-abundant miRNAs specifically expressed in the embryo or endosperm during grain development (Figure 7C). For example, miR156a specifically accumulated in the embryo and gradually increased from 15 to 28 DAP. In Arabidopsis embryos, miR156 delays the production of maturation transcripts by directing the repression of *SPL10/11*[66]. Therefore, wheat miR156 might also be involved in late embryo maturation during wheat grain development.

### The 22 nt miRNA regulation pathway in seed development

The present study showed that 22 nt miRNAs displayed markedly higher expression level in seed tissues, including in dry grains, germinating seed embryos, and grains 8 and 15 days after pollination, compared to other tissues, which was quite contrary to the expression pattern of 21 nt miRNAs (Figure 2B). It has been reported that 22 nt miRNAs and siRNAs are associated with AGO1, which recruits the RNA-dependent RNA polymerase RDR6 to generate double-stranded RNA from 3′ cleavage fragments, initiating the production of a second wave of siRNAs, referred to as secondary or “transitive” siRNAs [67]. We speculate that 22 nt miRNAs might involve in directing the generation of phased siRNAs during wheat seed germination and maturation. Enrichment of 22 nt sRNAs in grain has been reported in maize [28,47,48] and soybean [13], but not in rice [50,51]. The available data suggest that a different selection of 22 nt siRNAs/miRNAs involved in seed development might have arisen during the evolution of dicotyledon and monocotyledon plants. The accumulation of 22 nt miRNAs might be optimized to simultaneously silence multiple members of a gene clade, and ta-siRNAs triggered by 22 nt miRNAs might serve as a means to extend the targeting range of the primary miRNA [67]. In this study, northern blotting analysis revealed that two 22 nt miRNAs, tae-miR021b and tae-miR2003a, showed preferential accumulation in the embryo rather than the endosperm, suggesting important roles for miRNA-mediated gene regulation in wheat grain.

## Conclusion

We conducted a genome-wide survey of wheat miRNAs from multiple wheat tissues of different developmental stages. The results indicated that a total of 323 novel miRNAs were characterized and 366 previously reported miRNAs were confirmed in our dataset. Furthermore, 524 potential targets for 124 miRNA families were determined through degradome sequencing. Based on the genome-wide identification and characterization of miRNAs and their associated target genes, we further identified 64 miRNAs preferentially expressing in developing or germinating grains, which could play important roles in grain development.

## Methods

### Plant materials

Eleven tissues of the hexaploid Chinese Spring wheat (*Triticum aestivum* L.) cultivar were employed as a source for generating small RNA libraries. Dry grains were used without any treatment. Embryos of germinating seeds and shoots were dissected from seeds soaked in a Petri dish covered with a layer of filter paper saturated with water for 12 hours and approximately 3 days, when leaves were just at the coleoptile tip. Seedling leaves and roots were obtained from seedlings growing in a growth chamber under a relative humidity of 75% and 26/20°C day/night temperatures, with a light intensity of 3000 lx when the third leaf was at least 50% emerged. To collect stems at the jointing stage as well as young spikelets, flag leaves and developing grains, plants were grown in field conditions. Young spikelets were collected when they reached 0–5 mm and 10–15 mm in length, and grains were collected at 8 and 15 days after pollination (DAP). Flag leaves were cut, and spikes were labeled at the beginning of flowering during the principal flowering stage.

### RNA extraction, small RNA cloning and degradome library construction

Total RNA was isolated from frozen leaves using the TRIzol reagent (Invitrogen, USA) according to the manufacturer’s instructions. Low molecular weight RNA was enriched through precipitation with 0.5 M NaCl and 10% PEG8000. Approximately 100 μg of low molecular weight RNA was separated on a denaturing 15% polyacrylamide gel. RNA fragments with lengths between 18 and 26 nt was excised, purified from the gel, ligated to adaptors, reverse transcribed and subjected to PCR amplification. Approximately 100 μg of total RNA isolated from germinating seed embryos, seedling leaves, seedling roots and grains 8 days after pollination was used for degradome library construction, as described previously [20,21]. Small RNA and degradome libraries were sequenced using the Illumina GA IIx platform (BGI at Shenzhen).

### Identification of wheat miRNAs

The workflow for wheat miRNA identification (Additional file 8: Figure S3): The adaptor sequences were trimmed from the Illumina reads using ‘vector strip’ in the EMBOSS package. Reads with a length of 18–26 nt were mapped to the all of the available genome sequences including 454 reads with a 5X depth of coverage in the hexaploid wheat genome (http://www.cerealsdb.uk.net/CerealsDB/Documents/DOC_CerealsDB.php) [40], next-generation sequencing data of flow-sorted individual chromosome arms, provided by International Wheat Genome Sequencing Consortium (IWGSC) (http://www.wheatgenome.org/) and to wheat ESTs from the NCBI database and ESTs and cDNAs from the wheat genetic resources database (http://www.shigen.nig.ac.jp/wheat/komugi/ests/tissueBrowse.jsp;jsessionid=DD38CC8D511C04ADC414B40E0907544D.lb1) using the Bowtie package, version one [68]. Only perfectly matched sRNAs were used for further analysis.

The wheat genome is estimated to be composed of approximately 80% repeats, and the degradation of larger RNA molecules, such as rRNAs, would contaminate the sRNA libraries. Therefore, to remove the sRNAs originating from sequences such as repeats, rRNAs, or tRNAs, any sequences with matching hit counts in the 5X coverage wheat genome over 500 as well as those that mapped perfectly to non-coding RNAs in the Rfam database (http://rfam.sanger.ac.uk/) and repetitive sequences stored in Plant Repeat Databases (http://plantrepeats.plantbiology.msu.edu/downloads.html) were considered repeat-, rRNA- or tRNA-associated siRNAs [39]. The remaining clean sRNAs were subjected to miRNA identification using the modified package miReap, version 0.2. In the original version, minimum matched base pairs should be 14, which was revised to that mismatched base pairs was less than 4. MiRNA candidates with lengths of 20 nt to 24 nt and more than 20 reads in one library were used for the following analysis. The following key criteria were used for miRNA prediction: 1) the miRNA and miRNA* were derived from opposite stem-arms such that they formed a duplex with two-nucleotide 3′ overhangs; 2) the base-pairing between the miRNA and the other arm of the hairpin, which included the miRNA*, was extensive, such that there were typically four or fewer mismatched miRNA bases; 3) any asymmetric bulges were minimal in size (one or two bases) and frequency (typically one or less), especially within the miRNA/miRNA* duplex [69]; 4) the main miRNA sequence tag must cover at least 70% of all reads surrounding the miRNA start site from 20 nt upstream to 20 nt downstream of the site; and 5) the number of miRNA reads should be greater than 5 in either library.

The genome locus of miRNA precursors were determined through next-generation sequencing data of flow-sorted individual chromosome arms, provided by International Wheat Genome Sequencing Consortium (IWGSC) (http://www.wheatgenome.org/).

### Wheat miRNA evolution and conservation analysis

All wheat mature miRNAs were searched against genome sequences of other species to check whether these miRNAs exist in other species, which included the Arabidopsis genome, version 10.0 (http://www.Arabidopsis.org/), Rice genome, version 7.0 (http://rice.plantbiology.msu.edu/), maize genome 5b.60 (http://www.maizesequence.org/index.html), *Brachypodium distachyon* genome (ftp://brachypodium.org/brachypodium.org/Assembly/), barley genome (http://150.46.168.145/gbrowse_new/), soybean genome and sorghum genome (http://www.phytozome.net/) using bowtie version 1. The sequences surrounding a miRNAmatching site (200 bp upstream and downstream) from the other species were extracted and checked using a modified version of miReap0.2 (http://mireap.sourceforge.net). The miRNAs for which no precursor could be found in any other genome were considered wheat-specific miRNAs.

### Wheat miRNA target identification

We merged all of the wheat ESTs into a single wheat transcript dataset from NCBI, NCBI GEO database (GSE38344), EBI (ERP001415). A modified version of CleaveLand2 [70] was used to find the potential targets of all of the wheat miRNAs supported by our sRNA libraries with an alignment score of no more than 4.5 and at least 5 degradome reads validating the miRNA-induced cleavage site in the transcript. Additionally, to examine the locations of cleavage sites, GETORF [71] was used to find all ORFs longer than 70 amino acids. The locations of the cleavage sites were determined according to the relationship of the cleavage site with the start and end positions of the ORF. The target genes were classified into MapMan functional categories after searching for homologs among the MapMan categories found in TAIR9 using blastn. To map the cleavage sites of the target transcripts, we performed RNA ligation-mediated (RLM) rapid amplification of 5′cDNA ends using a modified GeneRacer kit protocol (Invitrogen).

### MiRNA expression analysis

Low molecular weight RNA (30 μg) was loaded into the lanes of a denaturing 15% polyacrylamide gel, resolved, and then transferred electrophoretically to Hybond-N + membranes (Amersham Biosciences, Buckinghamshire, UK). The membranes were UV crosslinked and baked for 2 hours at 80°C. DNA oligonucleotides complementary to miRNA sequences were end-labeled with γ-32P-ATP using T4 polynucleotide kinase (TaKaRa, Dalian, China). The membranes were prehybridized for more than 8 hours and then hybridized overnight in Church buffer at 38°C. Next, the blots were washed three times (two times with 2 × SSC + 1% SDS and one time with 1 × SSC + 0.5% SDS) at 50°C. Finally, the membranes were briefly air dried and then exposed to X-ray film for autography at -80°C. Images were acquired by scanning the films with FluorChem™ (Alpha Innotech, San Leandro, CA, USA).

SYBR® PrimeScript miRNA RT-PCR Kit were performed as manufacture’s instruction (TaKaRa). Briefly, 1 μg of total RNA was incubated with 10 μL of 2 × miRNA Reaction Buffer Mix (for Real Time), 2 μL of 0.1% BSA, 2 μL of miRNA PrimeScript RT Enzyme Mix, and 5 μL of RNase Free dH2O in a 20-μL reaction mixture. The temperature program was adjusted to run for 60 min at 37°C, 5 s at 85°C, and then 4°C forever. qRT-PCR was conducted on a Bio-Rad CFX96TM Real-Time System. Each reaction included 2 μL of product from the diluted RT reactions, 1.0 μL of miRNA primer (10 μM), 12.5 μL of SYBR Premix Ex Taq II(2×), and 8.5 μL of RNase Free water. The reactions were incubated in a 96-well plate at 95°C for 30 s, followed by 40 cycles of 95°C for 5 s, 59°C for 30 s, and 72°C for 30 s. All reactions were run in three replicates for each sample. The actin gene (GB#: AB181991) was used as the endogenous control. All of the probes and primers used in these analyses are listed in Additional file 9: Table S6.

### Availability of supporting data

The data set including the raw sequencing data of 11 small RNA libraries and 4 degradome libraries in our study are available in SNBI SRA database under accession no (accession: SRP040143) (http://www.ncbi.nlm.nih.gov/sra/?term=SRP040143). The sequences of mature miRNA, miRNA* and precursors, as well as the precursor genome location and secondary structure are available in Additional file 2: Table S2. The target genes obtained in this study are available in Additional file 4: Table S4. MiRNA expression by high throughput sequencing along 11 tissues is available in Additional file 6: Table S5.

## Competing interests

The authors declare that they have no competing interests.

## Authors’ contributions

FS performed experimental work and contributed bioinformatic analyses. GG and JD performed experimental work. WG performed planting of materials. HP and ZN contributed reagents and advice. QS and YY guided all aspects of the project and wrote the article. All authors read and approved the final manuscript.

## Supplementary Material

Additional file 1: Table S1 All of the reported known miRNAs in wheat. Click here for file

Additional file 2: Table S2 Precursors of novel miRNA and novel variants of known miRNAs in wheat. Click here for file

Additional file 3: Table S3 Variants of conserved miRNA in wheat and wheat specific miRNAs. Click here for file

Additional file 4: Table S4 Targets of conserved and wheat specific miRNAs. Click here for file

Additional file 5: Figure S1 miR159 non-conserved target Proline-, glutamic acid- and leucine-rich protein gene was identified by degradome sequencing. Click here for file

Additional file 6: Table S5 MiRNA expression along 11 tissues by high-throughput sequencing. Click here for file

Additional file 7: Figure S2 The normalized expression of highly conserved miRNAs was significantly higher than that of wheat-specific miRNAs. Click here for file

Additional file 8: Figure S3 Workflow for wheat miRNA and target gene prediction. Click here for file

Additional file 9: Table S6 All of the probes and primers used in this paper. Click here for file
