# Supplementary figures and images for: Whole-genome discovery of miRNAs and their targets in wheat (Triticum aestivum L.)
Source: BMC Plant Biol. 2014 May 22;14:142. doi: 10.1186/1471-2229-14-142 (PMC4048363; doi:10.1186/1471-2229-14-142)

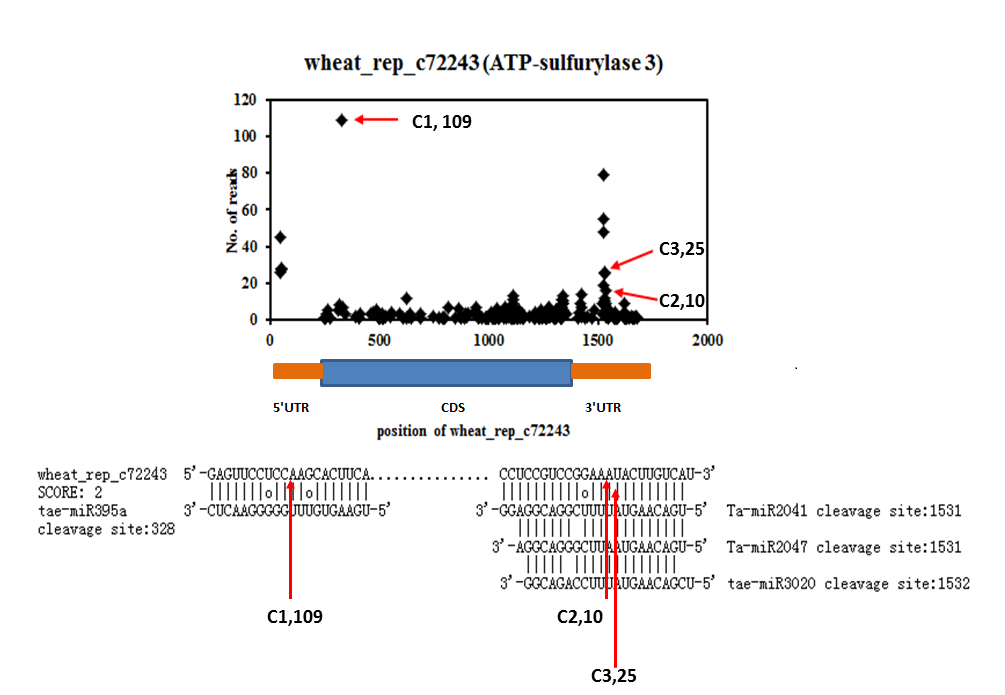

Supplement: Additional file 5: Figure S1 — miR159 non-conserved target Proline-, glutamic acid- and leucine-rich protein gene was identified by degradome sequencing. [file 1471-2229-14-142-S5.tiff]

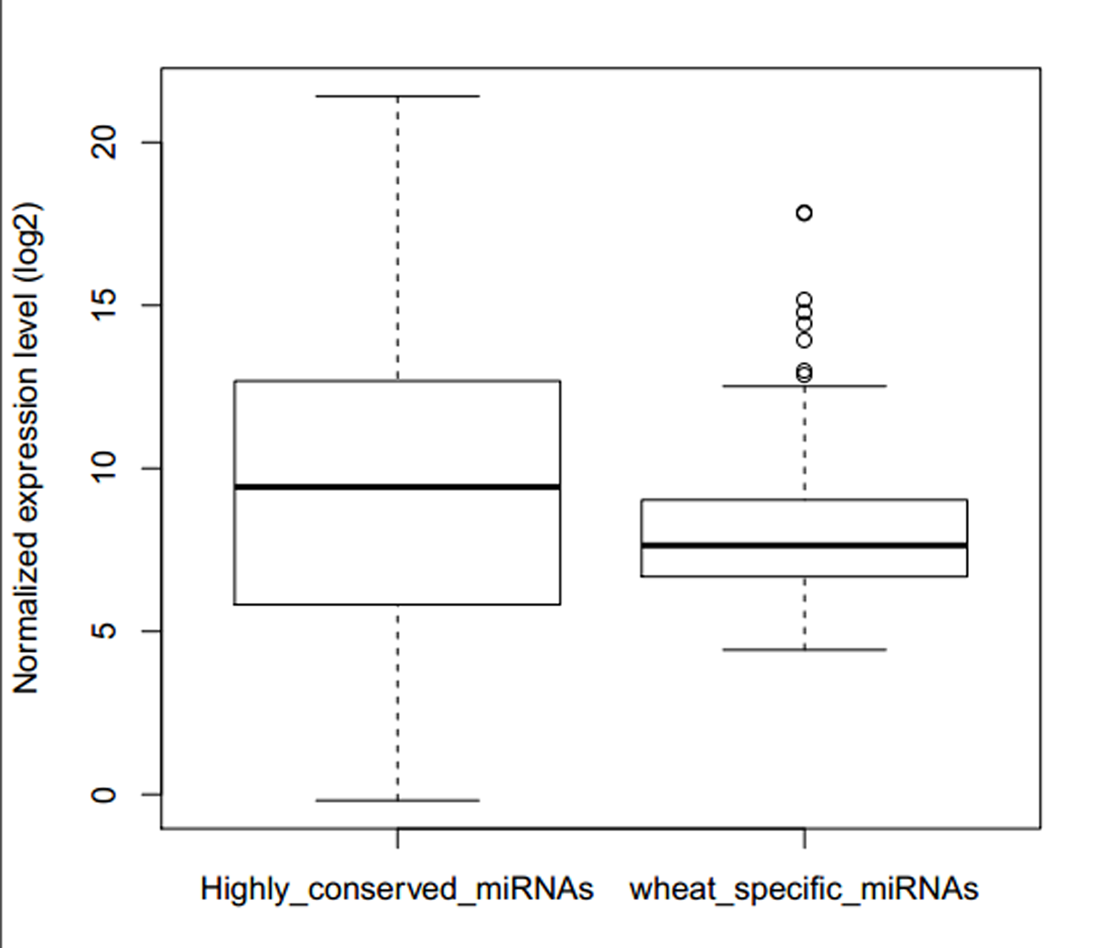

Supplement: Additional file 7: Figure S2 — The normalized expression of highly conserved miRNAs was significantly higher than that of wheat-specific miRNAs. [file 1471-2229-14-142-S7.tiff]

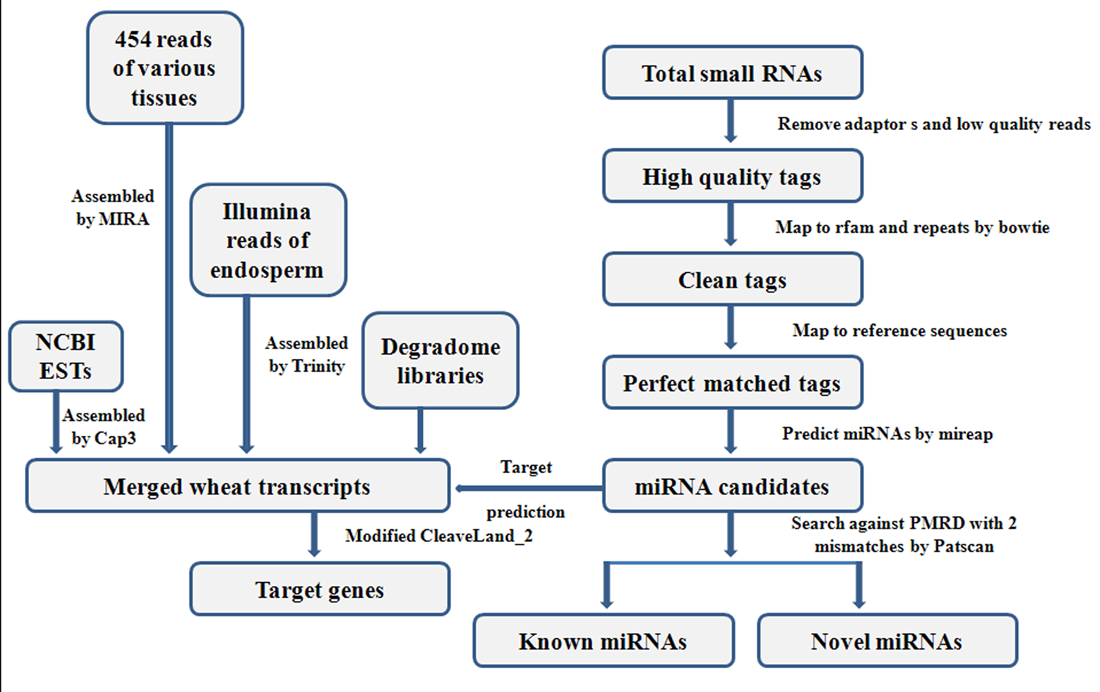

Supplement: Additional file 8: Figure S3 — Workflow for wheat miRNA and target gene prediction. [file 1471-2229-14-142-S8.tiff]
